# Turnbull procedure—analysis of a cohort in the salvage setting

**DOI:** 10.1007/s00384-026-05156-0

**Published:** 2026-05-28

**Authors:** Gerrit Arlt, Fabian Doyon, Richard Magdeburg, Peter Kienle

**Affiliations:** 1 General and Visceral Surgery, Brüderklinikum Julia Lanz, Mannheim, Germany; 2 General, Visceral, and Transplantation Surgery, Heidelberg University Hospital, Heidelberg, Germany

**Keywords:** Turnbull procedure, Delayed coloanal anastomosis, Hostile pelvis, LARS score, Fistula salvage

## Abstract

**Purpose:**

The Turnbull procedure (coloanal pull-through with delayed coloanal anastomosis, DCAA) serves as a salvage option for complex, therapy-refractory pelvic floor disorders to avoid permanent colostomy. This study evaluated the perioperative outcomes, stoma avoidance, and functional results of 16 patients treated between 2018 and 2024.

**Methods:**

A retrospective analysis of 16 consecutive patients with hostile pelvis (e.g., post-surgical fistulas, chronic pelvic sepsis, and Crohn’s disease). The key outcomes were time to anastomosis, Clavien-Dindo complications, stoma reversal rate, SF-12 quality of life, LARS, and Wexner continence score. Follow-up = 19–80 months.

**Results:**

Anastomosis occurred after a mean of 11 days (range, 6–19 days). Permanent stoma was avoided in 13/16 (81%) patients. Perioperative morbidity was low, and the SF-12 scores (14/16) ranged from 29 to 86%, correlating with the LARS (*p* = 0.016). In 12 patients, minor LARS was observed in 3/12, major LARS in 7/12, the Wexner incontinence score showed good continence in 4/12, moderate incontinence in 5/12, and severe incontinence in 3/12. The fistula subgroup showed the best functional results.

**Conclusions:**

The Turnbull/DCAA procedure enables sphincter preservation in complex pelvic disorders, particularly postoperative fistulas. Despite frequent major LARS and incontinence, 81% of the patients avoided permanent stoma. Meticulous selection and informed consent are essential because of the functional limitations.

**Supplementary Information:**

The online version contains supplementary material available at 10.1007/s00384-026-05156-0.

## Introduction

Coloanal pull-through with interval anastomosis (delayed coloanal anastomosis, DCAA) is a technique originally conceived for the treatment of Hirschsprung’s disease in childhood [1, 2]. It is frequently referred to as the Turnbull-Cutait procedure after its original describers, Turnbull and Cutait [3]. In rectal carcinoma resection, this procedure has experienced a renaissance in recent years [4, 5] as an alternative to one-stage transanal ultra-low anastomosis. Improved techniques in minimally invasive surgery, such as transanal platforms, 3D technology, robotics, and new anastomosis techniques such as transanal transsection single stapled (TTSS) anastomosis, appear to be more promising for the treatment of rectal carcinoma at present [6, 7].

Another important indication for the Turnbull procedure is complex, often therapy-refractory pelvic floor disorders requiring surgical intervention. In this specific problem area, the Turnbull procedure represents a “salvage” option for patients who wish to avoid a permanent colostomy and for whom an ultra-low primary (i.e., one-stage) anastomosis would be problematic: healing disturbances with persistent fistula and abscess cavities following complicated pelvic surgeries such as rectal resections or prostatectomies (“chronic pelvic sepsis”) [8, 9], additionally, in protracted, often multiply pre-operated fistula diseases, [10–13] which led to the term “hostile pelvis” [14]. This means in particular fibrosis, adhesions, and obliterated planes from at least 2 prior pelvic surgeries. A special collective in the context of this study includes patients with Crohn’s disease (CD), chronic fistula disease, and supra-anal stenosis.

The procedure consists of two parts: first, the residual rectum or diseased rectal portion and the fistula tissue are removed. This usually involves an intersphincteric (residual) proctectomy with sphincter preservation. In cases of fistulas, closure or reconstruction of the other fistula-bearing organs is also performed. The colon is mobilized abdominally, if possible, minimally invasively, until it can be pulled through the anus externally (“pull-through”). In contrast to the Soave procedure, the Turnbull-Cutait technique preserves the native anorectal mucosa. This avoidance of mucosal resection should minimize LARS risk by maintaining sensory and sphincter-supporting structures.

There are relatively few data in the literature regarding how many of the procedures were ultimately successful, that is, to what extent a definitive stoma could be avoided. Data on the functionality and quality of life (QoL) are scarce and almost exclusively refer to procedures performed primarily in the context of rectal carcinomas. Overall, this specific procedure is rarely performed for this indication.

### Objectives

This was a non-interventional, retrospective study of patients who underwent the Turnbull procedure (DCAA) at our institution between 2018 and 2024. In addition to perioperative parameters and evaluation of success in terms of avoiding a permanent stoma, special emphasis was placed on QoL and functional outcomes in the patients.

## Methods

This is a non-interventional, retrospective cohort study of consecutive patients treated with the Turnbull procedure (DCAA) at our institution between 2018 and 2024 (recruitment of *n* = 16 consecutive patients, Table 1). Patients were recorded in a database, and REDCap [15] was used for data processing. A positive vote was obtained from the Ethics Committee of Heidelberg University (reference no. S-526/2024). The STROBE guidelines were followed for reporting the results of this study.

**Table 1:** List of patients with indication/baseline diagnosis

Pt. no	Indication	No. of previous surgeries	Success, ileostoma reversal
1	Persistent fistula/abscess cavity after anastomotic insufficiency (AI) following low anterior rectal resection (LAR)—“chronic pelvic sepsis”	3	No
2	CD with supra-anal stenosis, perianal fistula	6	yes
3	CD with supra-anal stenosis, perianal fistula	3	yes
4	Persistent fistula/abscess cavity after LAR, recto-urethral fistula	3	yes
5	Recto-urethral fistula following prostatectomy	2	yes
6	Persistent abscess cavity presacral after AI following LAR—“chronic pelvic sepsis”	3	yes
7	CD with recto-vaginal fistula	8	no
8	Recto-vesical fistula following iatrogenic perforation of the bladder, Urothel-, bladder carcinoma	13	yes
9	Rectal prolapse re-re-recurrence	3	no
10	Recto-vaginal fistula following multiple gynecological surgeries	4	yes
11	Recto-vesical and recto-urethral fistula following failed restoration of continuity (diverticulitis)	2	yes
12	Persistent abscess cavity, AI following LAR—“chronic pelvic sepsis”	2	yes
13	Recto-vaginal fistula, AI following LAR (diverticulitis)	6	yes
14	Persistent abscess cavity und transsphincteric anal fistula, AI following LAR with resection of a recurrent dermoid tumor—“chronic pelvic sepsis”	12	yes
15	CD, supra-anal stenosis	2	yes
16	Persistent abscess cavity after AI following LAR—“chronic pelvic sepsis”	3	yes

All surgeries were performed at the Brüderklinikum Julia Lanz, Mannheim, Germany. A retrospective analysis was performed between December 2025 and January 2026. So QoL and functional outcomes were assessed via a one-time cross-sectional survey, without preoperative baselines or serial measurements.

**Fig. 1:**
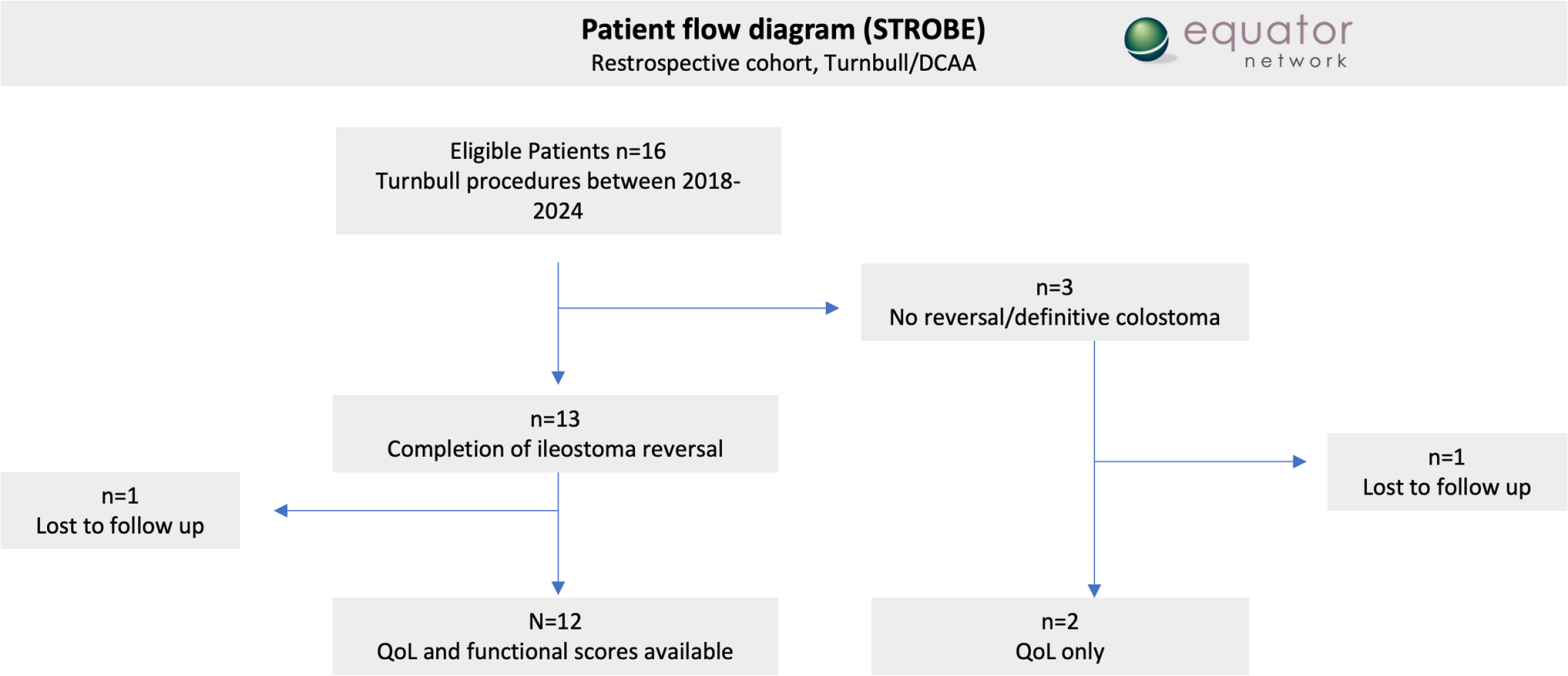
Patient flow chart

Follow-up: The time in days until delayed anastomosis, perioperative complication rate graded by the Clavien-Dindo classification, and rate of patients who were successfully treated, that is, avoidance of a definitive stoma to date, were recorded. A subgroup consisted of patients with defined fistula disease. QoL and functionality follow-up data (secondary outcomes) were collected throughout 2025, when the questionnaires were collected by mail, e-mail (survey function RedCap), or during outpatient visits. Quality of life (QoL) was assessed using the SF-12 questionnaire [16], continence function according to the Cleveland Clinic Incontinence Score (Wexner Score, CCS) [17], and the severity of Low Anterior Resection Syndrome (LARS) [18]. Baseline predictors included underlying diagnoses (e.g., post-surgical, defined fistulas such as recto-urethral/vesical/vaginal, chronic pelvic sepsis after anastomotic leak, and CD), multiple prior surgeries (“hostile pelvis”), and subgroups such as defined fistula disease (without CD). Confounders may include prior surgical history (multiple interventions), underlying disease severity (e.g., CD vs. post-surgical sepsis), follow-up duration (19–80 months), and response rates to questionnaires. Heterogeneity in indications (defined fistulas vs. chronic pelvic sepsis vs. CD) and small sample size (*n* = 16) could confound associations such as the SF-12 correlation with LARS. The consecutive recruitment of all 16 patients undergoing Turnbull/DCAA (2018–2024) at a single center minimized selection bias by avoiding cherry-picking. The STROBE guidelines were followed for the transparent reporting of methods and patient flow (Fig. 1).

There were no explicit adjustments for confounding (e.g., via multivariable analysis); a simple Spearman correlation was used for the SF-12/LARS/CCS due to the small, non-parametric sample size. Statistical calculations and boxplot visualizations were performed using the online data analysis program numiqo [19]. Post hoc subgroups were created for exploratory comparison: defined fistula (*n* = 7, non-CD; 100% healing, better CCS trends) versus CD (*n* = 4) versus “chronic pelvic sepsis” (*n* = 5). Groupings based on baseline diagnoses (Table 1) were used to explore the effects of etiology. Perplexity, an AI tool, was used for translation.

## Results

The cohort comprised 16 patients. The diagnoses included, alongside complicated courses following interventions on the rectum, prostate, and vagina, four patients with longstanding CD (Table 1).

All patients had undergone prior surgeries ex domo. A permanent stoma was avoided in 13 of the 16 patients. Fourteen could be followed up in the sense of completing the QoL questionnaires, and 12 completed the functional questionnaire (patient flow chart, Fig. 1). One patient in the no reversal/definitive stoma group did not answer due to a non-related illness, and one patient in the reversal/success group did not answer the questionnaires.

### Reasons for failure

Patient 1 experienced recurrent pelvic abscesses. Despite multiple attempts at interventional drainage, healing was not achieved. Ultimately, a proctectomy and the creation of a descendostomy was performed.

In the case of Patient 7, healing of the recto-vaginal fistula was accomplished; however, a new perianal/anastomotic fistula developed. Although this fistula was successfully treated, severe sphincter insufficiency precluded stoma reversal, and a definitive stoma has not yet been performed.

Patient 9 encountered recurrent prolapse of the neorectum. Following the unsuccessful attempt at treatment with sphincteroplasty, a proctectomy was conducted, and a descendostomy was established.

Follow-up was performed after a minimum of 19 months and a maximum of 80 months after the turnbull procedure (one-off cross-sectional evaluation). Anastomosis was performed on the median after an interval of 11 days (range, 6–19 days). Perioperative morbidity was low (Table 2).

**Table 2:** Perioperative morbidity (Clavien-Dindo)

Grade	Cases	Percentage
0 (no complications)	12	75%
I	2	12.5%
II	1	6.3%
IIIa	1	6.3%
IIIb–V	0	0%

SF-12 scores (FU in 14/16) ranged from 29 to 86%, with poor quality of life values predictably correlating with poor LARS scores (Spearman correlation analysis, *p* = 0.016), but not significantly with CCS (*p* = 0.333). Functional scores were obtained in 12 patients: no LARS occurred in 3/12 (25%), minor LARS was present in 2/12 (16.6%), and major LARS in 7/12 (58.3%). According to the Wexner score, 4/12 patients showed good continence (CCS < 10, 33%). Five out of 12 patients suffered from moderate (> 10, < 15, 42%) and 3/12 from severe incontinence (25%), respectively (Table 3).

**Table 3:** Functional results and quality of life

Pt. no	SF-12 Score	LARS Score	Wexner Score (CCS)
1	86%	AP	AP
2	74%	25	14
3	69%	32	13
4	40%	31	9
5	83%	16	9
6	77%	32	16
7	74%	AP	AP
8	83%	21	4
9	LTFU	AP	AP
10	29%	41	13
11	71%	9	1
12	LTFU	LTFU	LTFU
13	86%	31	13
14	37%	37	17
15	51%	34	15
16	86%	20	11

The subgroup of patients with defined fistula disease (excluding patients with CD) included seven patients (Table 4): Complete closure was achieved in all cases; however, redo procedures were required in two patients (1 × gracilis plasty and 1 × direct closure).

**Table 4:** Patients with defined fistulas

Pt. no	Diagnosis	Fistula healing
4	Recto-urethral fistula	Yes
5	Recto-urethral fistula	Yes
8	Recto-vesical fistula	Yes
10	Recto-vaginal fistula	Recurrence, healed with gracilis plasty
11	Recto-urethral and -vesical fistula	Yes
13	Recto-vaginal fistula	Recurrence, healed after redo (direct closure)
14	Trans-sphincteric anal fistula	yes

**Fig. 2:**
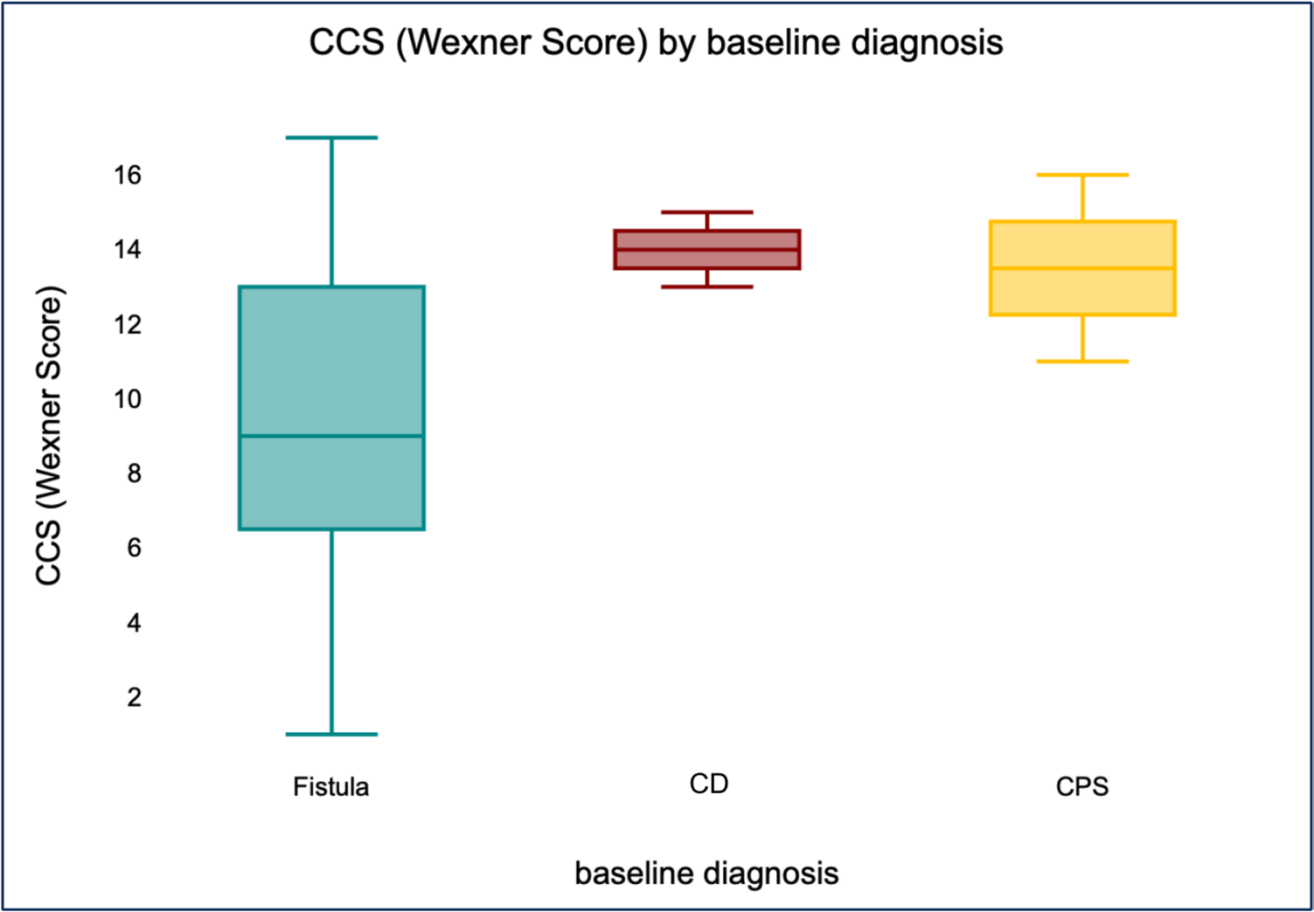
Boxplot representation of the distribution of CCS values by initial condition (numiqo.de). CD Crohn’s disease, CPS chronic pelvic sepsis

In these patients, QoL and functional scores tended to be better, although no statistical significance was demonstrated in this small differentiated group. The numerical difference was most pronounced in the incontinence score (CCS; Fig. 2). All patients in this group completed the questionnaire and were able to avoid permanent stomas.

In the three CD patients with defined fistulas, permanent closure could not be achieved. Although the recto-vaginal fistula healed, an anal fistula recurrence occurred. Nevertheless, both patients who presented with an anal fistula as the initial finding were able to avoid a permanent stoma to date despite recurrences and ongoing need for therapy, whereas the patient with the healed recto-vaginal fistula could *not* due to the new anal fistula (Table 5).

**Table 5:** CD patients with fistulas

Pt. no	Diagnosis	Recurrence
2	Anal fistula	Yes, 5× redo surgery
3	Anal fistula	Yes, redo surgery, therapy with stem cells
7	Recto-vaginal fistula	Rekto-vaginal fistula healed, but new perianal fistulas

## Discussion

This retrospective analysis shows as a key result that the Turnbull procedure (DCAA) can be an effective salvage option for patients with highly complex pelvic floor pathologies. In a patient cohort characterized by high morbidity, multiple previous surgeries, and often longstanding disease courses (“hostile pelvis”), a permanent stoma could be successfully avoided in 13 of 16 patients (81%). This rate is remarkable, given the considerably high number of previous surgeries in these cases. In a study by Justiniano and Hull [8], this rate was only 50% at 5 years postoperatively. However, our success rate is within the range of previously published case series that have described similar indications [11, 20, 21]. Our series included more patients than those of Lavryk et al., although the follow-up periods were not entirely comparable.

Perioperative morbidity was low, underscoring that the two-stage technique can be performed safely. In particular, the low rate of severe complications (1 × Clavien-Dindo IIIa) demonstrates that DCAA can be performed safely—even in complex situations. The average interval of 11 days until definitive suturing is somewhat longer than the usual timeframe of the originally described Turnbull technique, which may be due to the difficult and chronically damaged tissues compared to DCAA, for example, in primary surgery for rectal carcinoma. Taking these chronic damages into consideration, the second step was postponed for a relatively extended period. However, the perfusion of the pulled-through colon and to a certain extent also patient comfort impose a practical limit on this delay.

### Fistula closure

The good results of fistula closure, particularly in recto-vaginal fistulas, appear reproducible [22, 23]. Primarily, DCAA was successful in five of seven patients, and one additional intervention was performed in each patient. Here, satisfaction is comparatively high, and the functional scores are also in the upper range. In contrast, the fistula healing rate in CD’s is predictably poor, although this group in the cohort is very small.

### Functional results

Another result of this study is the functional data, which have rarely been described in the literature, mostly in the context of rectal cancer surgery. Despite the high risk of functional limitations due to prior surgeries, scar formation, and sphincter damage, the patients showed partially good functional results: four of the 12 assessed patients achieved a low Wexner score, and five met the criteria for no or minor LARS. Nevertheless, it must be noted that a significant proportion exhibited relevant functional limitations (major LARS in 7/12, severe incontinence in 3/12). However, these results must be interpreted against the background of the initial situation: in many cases, no sphincter-preserving procedure would have been possible without DCAA involvement. Moreover, the observed limitations correspond in severity to the functional outcomes described for ultra-low anastomoses in carcinoma surgery [24].

### Quality of life

Notably, the wide range of SF-12 scores reflects significant interindividual variation in the perceived QoL. The correlation between low SF-12 scores and pronounced LARS confirms that functional limitations after DCAA are major determinants of QoL. Nevertheless, individual patients with acceptable functional outcomes report a good health-related quality of life (QoL). This indicates that satisfactory functional reconstruction is possible, even for complex pathologies. Here, the baseline situation in this specific cohort must be considered, as patients often suffer from this issue for years beforehand. Another portion of patients continues to suffer, or has begun suffering again, from the underlying disease, e.g., in the context of tumor recurrence elsewhere, which naturally influences the current SF-12 value.​

The main limitations of this study are its small sample size and retrospective design. The heterogeneity of the patient cohort regarding underlying diseases and prior surgeries also complicates direct comparisons with other techniques and subgroup analyses. Whether the small CD subgroup particularly benefits remains unclear, as fistula recurrences occurred in 3 of 4 patients, yet a stoma-free perspective still existed, lending even greater significance to this markedly younger subgroup.​

An interesting aspect would be the baseline value for quality of life to enable a comparison with the follow-up results. However, given the highly complex disease courses before and after pull-through surgery in nearly all patients, establishing such a correlation would be difficult. Moreover, it must be noted that the quality of life with a permanent stoma, particularly a colostomy, is not necessarily poor. A functionally poor coloanal anastomosis certainly impacts daily life more negatively than a well-constructed anus praeter. This is confirmed here too: the two patients with definitive anus praeter who responded have relatively good SF-12 values.​

This study provides one of the most comprehensive insights to date into the functional outcomes of the Turnbull procedure in the salvage setting, outside oncologic rectal surgery. Ideally, prospective studies would examine functional long-term results on a larger scale; however, this is hardly feasible given the specific cohort and the demanding surgical technique. Overall, the results underscore that Turnbull/DCAA represents a valuable tool for sphincter-preserving reconstruction in complex pelvic floor disorder cases. This enables patients for whom a definitive stoma would otherwise be the only option to maintain anatomically correct continence. Above all, complex fistulas arising from intra- or postoperative complications strongly indicate DCAA. Given the functional limitations described, meticulous patient selection and informed consent are essential.

## Supplementary Information

Below is the link to the electronic supplementary material.

ESM 1 (DOCX 31.4 KB)

ESM 2 (PDF 6.88 MB)

## Data Availability

The data that support the findings of this study are available within the article, additionally clinical data is  not publicly available due to privacy and ethical restrictions but are available from the corresponding author upon reasonable request and ethics approval.
