# Supplementary material for: Turnbull procedure—analysis of a cohort in the salvage setting
Source: Int J Colorectal Dis. 2026 May 28;41(1):94. doi: 10.1007/s00384-026-05156-0 (PMC13219227; doi:10.1007/s00384-026-05156-0)
Supplement: Supplementary file 2 — (PDF 6.88 MB) [file 384_2026_5156_MOESM2_ESM.pdf]

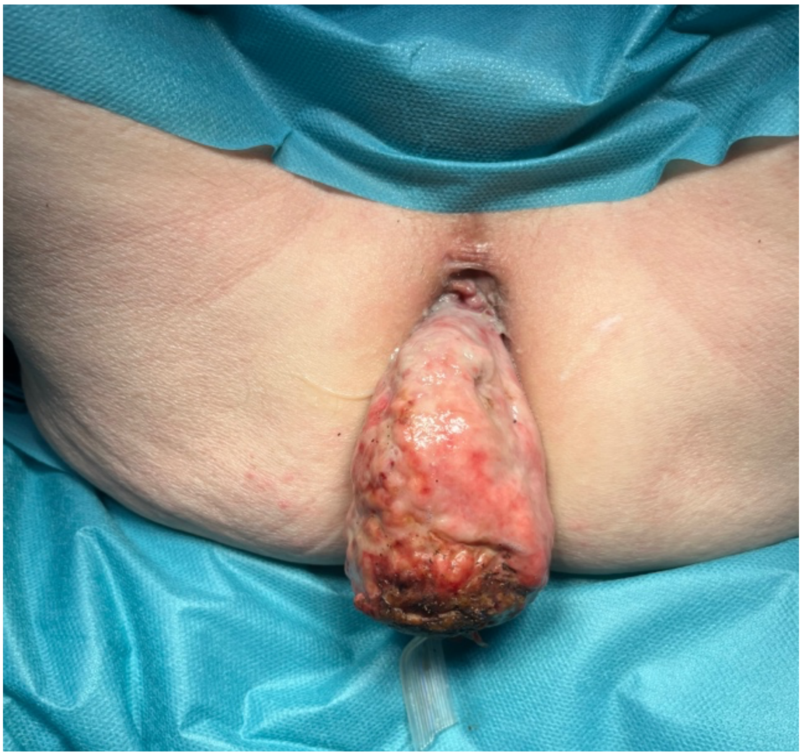

Figure 1: Stapled colon pulled through externally through the anus, EasyFlow drain inserted for decompression (8 days after first operation).

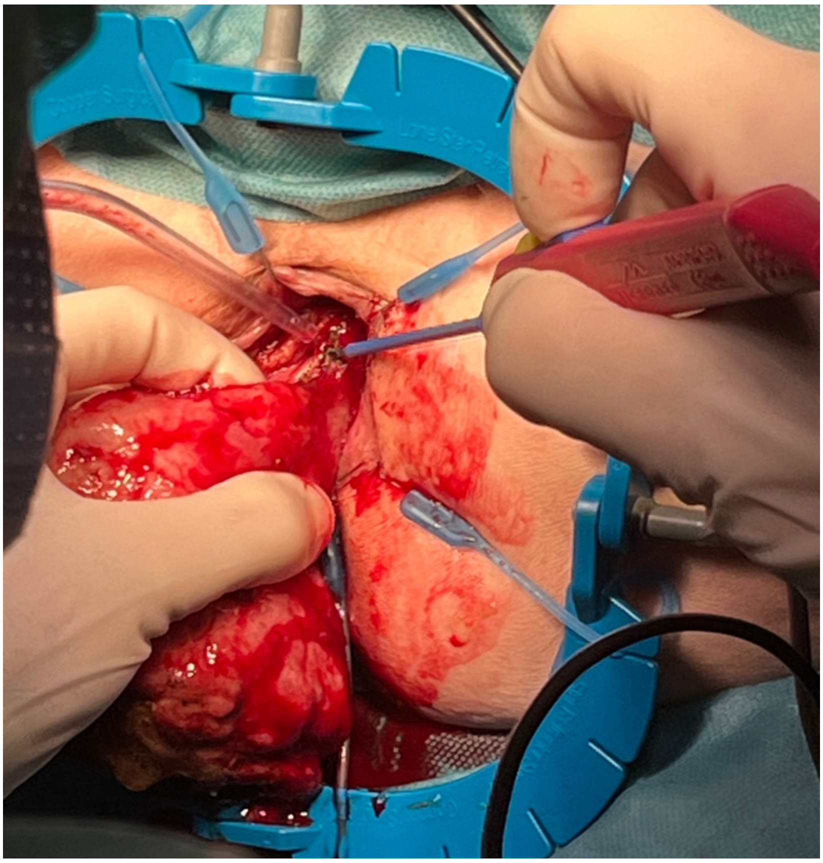

Fig. 2: Resection of the protruded colon-segment.

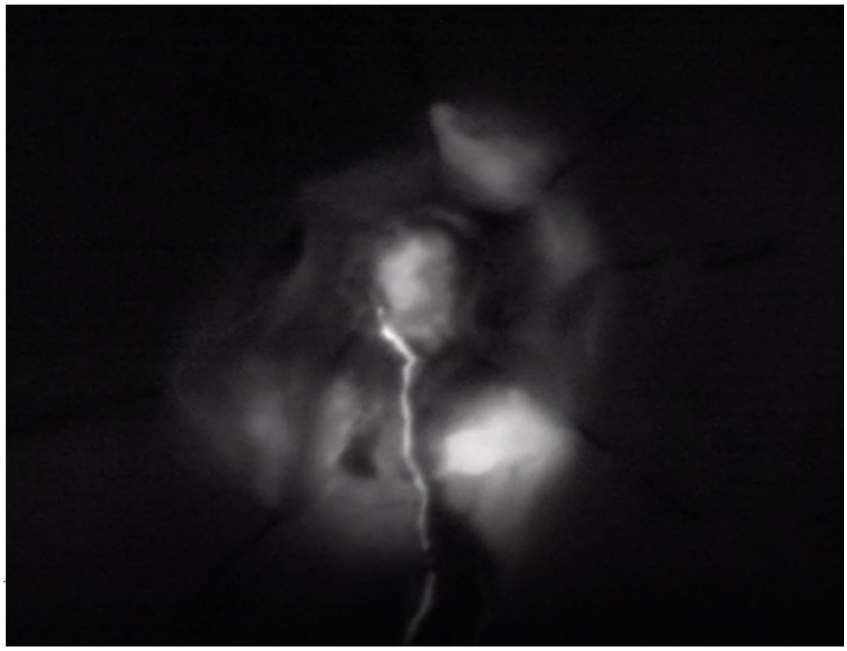

Fig. 3: Indycyan-green control of the colon-perfusion.

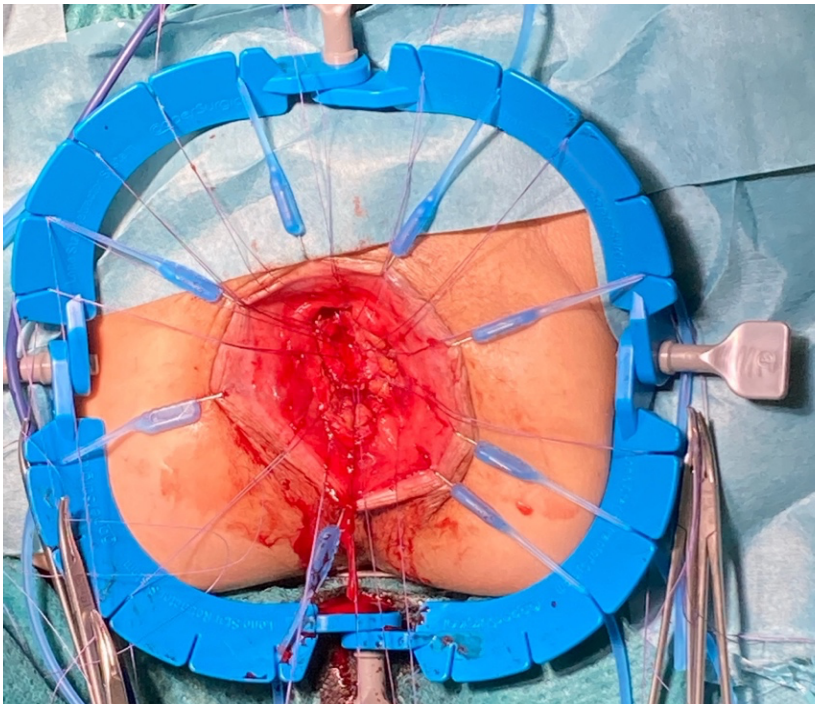

Fig. 4: Anastomosis with placed interrupted sutures (absorbable).

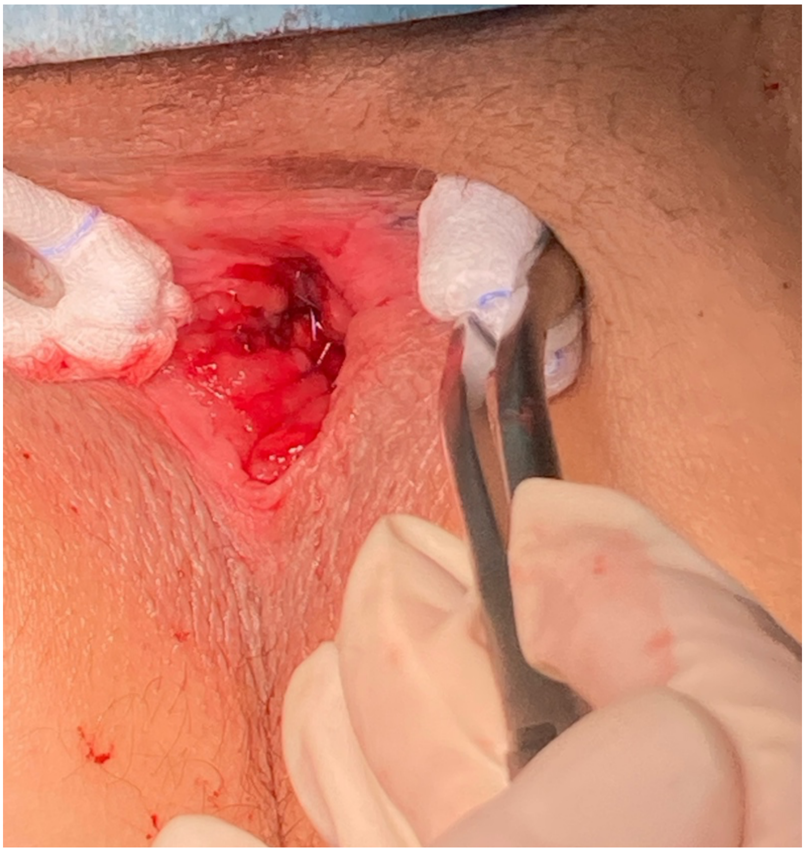

Fig. 5: Well retracted completed coloanal anastomosis after removal of the supporting frame.
